# Einfluss der COVID-19-Pandemie auf die physiotherapeutische und ärztliche Nachsorge nach Rekonstruktionen des vorderen Kreuzbandes

**DOI:** 10.1007/s00132-021-04197-3

**Published:** 2022-01-03

**Authors:** Benjamin Bartek, Tobias Jung, Antonia Schwiedernoch, Carsten Perka, Yannick Palmowski

**Affiliations:** Center for Musculoskeletal Surgery, Charité – Universitätsmedizin Berlin, Humboldt-Universität zu Berlin, Berlin Institute of Health, Chariteplatz 1, 10117 Berlin, Deutschland

**Keywords:** VKB-Rekonstruktion, „Coronavirus disease“ 2019, Kniegelenk, Physiotherapeuten, SARS-CoV‑2, ACL reconstruktion, “Coronavirus disease” 2019, Knee joint, Physical therapists, SARS-CoV‑2

## Abstract

**Hintergrund:**

Ziel der Studie war es, den Einfluss der COVID-19-Pandemie auf die physiotherapeutische und ärztliche Nachsorge nach Rekonstruktion des vorderen Kreuzbandes (VKB) zu untersuchen.

**Methoden:**

Insgesamt wurden 116 Patienten (72 Männer und 44 Frauen) eingeschlossen, die zwischen September 2019 und Dezember 2020 eine Rekonstruktion des VKB erhalten hatten. Die Patienten wurden in eine 2019 vor der COVID-19(„coronavirus disease“ 2019)-Pandemie operierte Patientengruppe sowie eine 2020 operierte, von der COVID-19-Pandemie unmittelbar betroffene Patientengruppe eingeteilt. Anhand eines standardisierten Fragebogens wurden die Auswirkungen der Pandemie auf die ärztliche und physiotherapeutische Nachsorge sowie auf die Dauer der postoperativen Arbeitsunfähigkeit erhoben. Ergänzend erfolgte eine Auswertung des Bewegungsumfanges des operierten Knies 3 Monate postoperativ.

**Ergebnisse:**

Es zeigte sich bei den 2020 operierten Patienten eine deutliche Tendenz zu einem höheren Anteil an Streckdefiziten ≥ 5° (18,8 % vs. 4,3 %, *p* = 0,097) oder einer maximalen Beugefähigkeit von < 120° (23,3 % vs. 10 %, *p* = 0,197) 3 Monate postoperativ, die allerdings nicht signifikant war. In der physiotherapeutischen und ärztlichen Nachsorge berichteten die 2020 operierten Patienten von signifikant längeren Wartezeiten auf Termine, späteren Zeitpunkten des ersten postoperativen Termins, einer höheren Anzahl vergeblicher Terminanfragen sowie einer höheren Anzahl abgesagter Termine. 34,9 % der 2020 operierten Patienten gaben an, die Dauer ihrer postoperativen Arbeitsunfähigkeit durch die Möglichkeiten zur Arbeit im Homeoffice verkürzt haben zu können. Pandemiebedingte alternative Behandlungsangebote wurden den Patienten von 13,3 % der Physiotherapeuten sowie 12,2 % der Ärzte angeboten.

**Schlussfolgerung:**

Obwohl die physiotherapeutische und ärztliche Behandlung von keinem „Lockdown“ betroffen war, kam es aufgrund der Pandemie zu signifikanten Einschränkungen, welche sich auch in einer deutlichen Tendenz zu schlechteren klinischen Ergebnissen niederschlagen. Es besteht daher Bedarf an einem weiteren Ausbau alternativer Therapieoptionen, die bislang nur von 12–13 % der Praxen zur Verfügung gestellt werden und mutmaßlich auch für andere Erkrankungen von ähnlicher Bedeutung sind.

## Abstract (en)

**Background:**

The aim of this study was to examine the influence of the COVID-19(coronavirus disease 2019) pandemic on the medical and physiotherapeutic follow-up care after reconstructions of the anterior cruciate ligament.

**Methods:**

116 patients (72 men and 44 women) who had received a reconstruction of the anterior cruciate ligament between September 2019 and December 2020 were included in this study. These patients were divided into two groups depending on the date of surgery: one group that had received surgery in 2019 before the COVID-19 pandemic and one group that had undergone surgery in 2020 and was directly affected by the COVID-19 pandemic. All patients were interviewed using a standardized questionnaire regarding the influence of the coronavirus pandemic on the medical and physiotherapeutic follow-up care, as well as the moment of return to work. In addition, the range of motion of the respective knee 3 months postoperatively was analysed based on physical examination.

**Results:**

At 3 months postoperatively, patients who had undergone surgery in 2020 showed a clear trend towards a higher frequency of extension deficits of ≥ 5° (18.8% vs. 4.3%, *p* = 0.097) or an inability to bend the knee ≥ 120° (23.3% vs. 10%, *p* = 0.197) compared to those who had received surgery in 2019. Patients who had undergone surgery in 2020 reported significantly longer delays for appointments, a higher number of futile attempts to get an appointment and a higher number of cancelled appointments, regarding both medical and physiotherapeutic follow-up care. 34.9% of the patients who received surgery in 2020 indicated that they were able to reduce the duration of their sick leave due to the increased possibilities of working in a home-office situation during the pandemic. Alternative treatment options due to the pandemic were offered by 13.3% of the physiotherapists and 12.2% of the physicians.

**Conclusion:**

Although the physiotherapeutic and medical follow-up care was not directly affected by a “lockdown”, the pandemic led to significant restrictions, which are also reflected in a clear trend towards worse clinical outcomes. Consequently, a further expansion of alternative treatment options, which were only offered by 12–13% of practices and that are presumably of comparable relevance for various other diseases, is needed.

## Einleitung

Mit einer jährlichen Inzidenz von über 40.000 Rupturen des vorderen Kreuzbandes (VKB) deutschlandweit sowie etwa 1/1000 in der Altersgruppe 15–25 Jahre gehört die VKB-Ruptur zu den häufigsten Sportverletzungen junger Patienten [1–3]. Insbesondere bei jungen Patienten steht laut aktueller S1-Leitlinie aufgrund des hohen funktionellen Anspruchs sowie des langfristig erhöhten Arthroserisikos bei VKB-Insuffizienz die operative Therapie im Vordergrund, die in der Regel durch eine Rekonstruktion mit autologer Sehnenplastik erfolgt [4, 5].

Für ein optimales postoperatives Outcome mit langfristig möglichst vollständiger Wiederherstellung der Funktionalität und Belastbarkeit ist die physiotherapeutische Nachsorge von entscheidender Bedeutung. Diese erfolgt üblicherweise entsprechend standardisierter Nachbehandlungsschemata. Die Relevanz einer konsequenten Physiotherapie mit frühzeitigem Beginn der Therapie, kontinuierlicher Wahrnehmung aller Termine und regelmäßiger Kontrolle des Fortschritts ist in Studien belegt [6, 7].

Seit Ende 2019 verbreitet sich die „coronavirus disease“ 2019 (COVID-19) weltweit. In Deutschland wurde zum Infektionsschutz ab dem 11.03.2021 an die Bevölkerung appelliert, Kontakte zu beschränken. Bereits kurz darauf trat ab dem 22.03.2020 der erste „Lockdown“ in Kraft und führte zu deutlichen Einschränkungen in vielen Bereichen des öffentlichen Lebens [8]. Bisher ist unseres Wissens ungeklärt, welche Auswirkungen die COVID-19-Pandemie sowie die damit verbundenen Kontaktbeschränkungen auf die postoperative Nachsorge hatten und auch die Folgen für das klinische Outcome sind bislang nicht bekannt. Um dies zu untersuchen, führten wir in der vorliegenden Studie daher eine Befragung von Patienten durch, welche während oder direkt vor der Pandemie eine Rekonstruktion des VKB erhielten und ergänzten dies durch postoperativ erfasste klinische Untersuchungsbefunde. Ziel war es, zu ermitteln, ob die im Rahmen der Pandemie getroffenen Maßnahmen einen Einfluss auf die physiotherapeutische und ärztliche Nachsorge nach VKB-Rekonstruktionen hatten, sowie ob es hierdurch zu einer Beeinträchtigung der klinischen Ergebnisse kam. Dabei sollte die VKB-Rekonstruktion in erster Linie als Beispiel für eine Vielzahl an orthopädischen und auch sonstigen Erkrankungen dienen, bei denen eine enge ärztliche und physiotherapeutische Betreuung erforderlich ist.

## Material und Methoden

### Ethikvotum

Die Durchführung der Studie wurde von der Ethikkommission der Charité – Universitätsmedizin Berlin genehmigt (EA2/072/21).

### Patienten

Für die Durchführung dieser retrospektiven Kohortenstudie wurden alle volljährigen Patienten kontaktiert, die im Zeitraum zwischen September 2019 und April 2020 am Centrum für Muskuloskeletale Chirurgie der Charité – Universitätsmedizin Berlin eine Rekonstruktion des VKB erhalten haben. Diese Patienten wurden je nach Operationszeitpunkt in zwei Gruppen eingeteilt: eine 2019 operierte Gruppe, welche mindesten 12 Wochen Physiotherapie vor Inkrafttreten des ersten „Lockdowns“ erhielt, sowie eine 2020 operierte Gruppe, welche während der ersten 3 postoperativen Monate von Einschränkungen durch die COVID-19-Pandemie sowie den dadurch bedingten „Lockdown“ betroffen war.

### Datenerhebung

Bei Einverständnis zur Studienteilnahme erfolgte eine telefonische Befragung der Teilnehmer sowie eine Auswertung der im Rahmen der postoperativen Nachkontrollen routinemäßig erfassten klinischen Untersuchungsbefunde. Im Rahmen der telefonischen Datenerhebung wurden die Teilnehmer anhand eines vorher definierten standardisierten Fragebogens zu ihren Erfahrungen bei der postoperativen physiotherapeutischen und ärztlichen Nachsorge in Zeiten der COVID-19-Pandemie befragt. Erfragt wurden dabei (jeweils separat für die ärztliche und physiotherapeutische Nachsorge) die Wartezeit auf einen Termin, der Zeitraum zwischen Operation und Wahrnehmen des ersten Termins, die Anzahl vergeblicher Terminanfragen, die Anzahl selbst oder durch die jeweilige Praxis abgesagter Termine, Einschränkungen der Behandlung durch die Pandemie, aufgrund der Pandemie eingeführte alternative Therapieoptionen, generelle Zufriedenheit mit der Behandlung (auf einer Skala von 1–10) sowie die Einschätzung des Einflusses der Pandemie auf die Qualität der Nachsorge (auf einer Skala von 1–10). Zudem wurde erfragt, wie lange die Patienten postoperativ krankgeschrieben waren, ob die Dauer der Arbeitsunfähigkeit aufgrund von Homeoffice im Rahmen der Pandemie verkürzt werden konnte und ob sich die Patienten erneut in einer vergleichbaren Situation operieren lassen würden. Ergänzend zu den telefonisch erhobenen Daten wurde bei allen Patienten der im Rahmen der routinemäßigen Nachkontrollen nach klinischer Untersuchung dokumentierte Bewegungsumfang (Neutral-Null-Methode) des operierten Knies 3 Monate postoperativ erfasst und es wurde der Anteil an Patienten mit einem Streck- oder Beugedefizit errechnet. Hierbei wurden ein Streckdefizit von ≥ 5° oder eine maximale Beugefähigkeit von < 120° als relevante Bewegungseinschränkung gewertet. Die Befragung und die Auswertung der klinischen Untersuchungsbefunde erfolgte im April 2021, sodass sich zu diesem Zeitpunkt alle Patienten bereits seit mindestens 12 Wochen in der postoperativen Nachbehandlung befanden.

### Statistische Auswertung

Die statistische Auswertung erfolgte mit dem Programm SPSS in der Version 27 (IBM, New York, NY, USA). Zur Untersuchung der Unterschiede zwischen beiden Gruppen auf Signifikanz wurden bei parametrischen Variablen der Student’s T‑Test, bei nichtparametrischen Variablen der Mann-Whitney-U-Test sowie bei binären Variablen der Chi-Quadrat Test genutzt. Für den Vergleich von > 2 Gruppen wurde eine einfaktorielle Varianzanalyse (ANOVA) durchgeführt.

## Ergebnisse

### Studienkollektiv

Insgesamt erhielten im Zeitraum zwischen September 2019 und Dezember 2020 168 Patienten eine Rekonstruktion des VKB, von denen 8 aufgrund von Minderjährigkeit ausgeschlossen wurden. Von den verbleibenden Patienten konnten 116 (72 Männer und 44 Frauen) kontaktiert werden und erklärten sich zur Studienteilnahme bereit. Von diesen wurden 33 (23 Männer und 10 Frauen) 2019 vor der COVID-19-Pandemie operiert, wohingegen 83 (49 Männer und 34 Frauen) Patienten 2020 operiert wurden und somit zumindest Teile der Nachsorge während der COVID-19-Pandemie absolvierten. Das Durchschnittsalter lag bei 33,9 Jahren (2019: 34 Jahre, 2020: 33,8 Jahre). Entnahmestelle für das Sehnentransplantat war in der Regel der M. semitendinosus. Daneben wurden in zwei Fällen (davon 1 im Jahr 2019 und 1 im Jahr 2020) die Sehnen des M. semitendinosus und M. gracillis sowie in 13 Fällen (davon 4 im Jahr 2019 und 9 im Jahr 2020) ein Transplantat aus der Quadrizepssehne genutzt. Alle Operationen während des untersuchten Zeitraums wurden von den gleichen drei erfahrenen Operateuren in einem stationären Setting durchgeführt. Die Nachbehandlung erfolgte anhand standardisierter Nachbehandlungsschemata, die während des untersuchten Zeitraums nicht verändert wurden.

### Auswirkungen auf die physiotherapeutische Nachsorge

Bei der Befragung zur physiotherapeutischen Nachbehandlung zeigten sich hinsichtlich aller erfragten Items signifikante Unterschiede zwischen den 2019 und 2020 operierten Patienten (Tab. 1).

**Table 1:** 

	2019		2020		*p*
	Mittelwert	SD	Mittelwert	SD	
Wartedauer auf Termin (Wochen)	1,09	0,29	2,57	5,75	0,022
Zeitpunkt des ersten Termins (Wochen postoperativ)	1,15	0,44	1,88	1,82	0,001
Anzahl vergeblicher Terminanfragen	0,26	1,14	0,94	1,52	0,010
Anzahl durch Physiotherapie abgesagter Termine	0,32	0,91	1,16	1,67	0,001
Anzahl selbst abgesagter Physiotherapietermine	0,12	0,41	0,90	1,72	0,000
Zufriedenheit mit der Behandlung (1 = sehr unzufrieden, 10 = sehr zufrieden)	8,56	1,16	7,83	1,66	0,021

*SD* Standardabweichung

### Auswirkungen auf die ärztliche Nachsorge

Bei der Befragung zur ärztlichen Nachbehandlung zeigten sich hinsichtlich der Fragen zu Terminvereinbarung und -wahrnehmung signifikante Unterschiede zwischen den 2019 und 2020 operierten Patienten (Tab. 2).

**Table 2:** 

	2019		2020		*p*
	Mittelwert	SD	Mittelwert	SD	
Wartedauer auf Termin (Wochen)	1,30	1,02	2,28	1,81	0,000
Zeitpunkt des ersten Termins (Wochen postoperativ)	1,42	1,28	2,38	1,81	0,002
Anzahl vergeblicher Terminanfragen	0,03	0,17	0,32	0,65	0,000
Anzahl durch Orthopäden abgesagter Termine	0,03	0,17	0,38	0,90	0,001
Anzahl selbst abgesagter Orthopädietermine	0,00	0,00	0,29	0,60	0,000
Zufriedenheit mit der Behandlung (1 = sehr unzufrieden, 10 = sehr zufrieden)	7,48	2,03	7,16	1,81	0,400

*SD* Standardabweichung

### Einfluss auf das postoperative Bewegungsausmaß

Hinsichtlich des Bewegungsumfanges 3 Monate postoperativ zeigte sich bei den 2020 operierten Patienten eine nicht signifikante Tendenz zu einem häufigeren Streckdefizit ≥ 5° als vor der COVID-19-Pandemie (4,3 % vs. 18,8 %, *p* = 0,097). Zudem bestand eine Tendenz zu einem häufigeren Beugedefizit mit einer maximalen passiven Beugefähigkeit von < 120°, welche ebenfalls nicht signifikant war (10 % vs. 23,3 %, *p* = 0,197).

### Einfluss der COVID-19-Pandemie auf die Dauer der Arbeitsunfähigkeit

**Figure 1:**
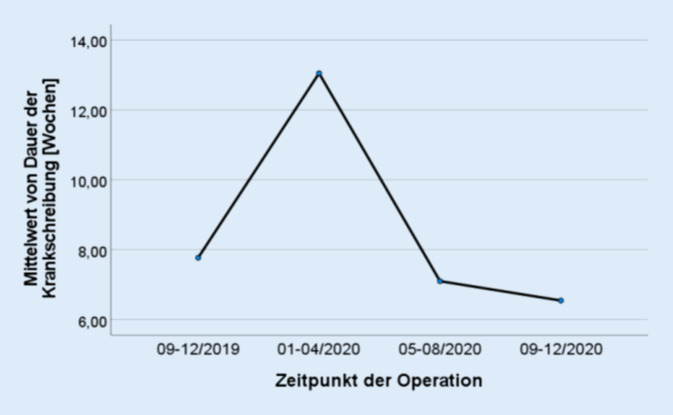

Einer von 33 Patienten 2019 (2,9 %) sowie 29/82 Patienten 2020 (34,9 %) gaben an, dass sich durch die Pandemie die Dauer ihrer postoperativen Arbeitsunfähigkeit verkürzt habe. Im Durchschnitt betrug die Dauer der Arbeitsunfähigkeit 2019 7,77 (±6,10) Wochen und 2020 8,53 (±10,37) Wochen (*p* = 0,688). Bei genauerer Unterteilung des Jahres 2020 in einzelne Tertiale zeigt sich ein signifikanter Unterschied in der Dauer der Arbeitsunfähigkeit mit 13,04 (±3,68) Wochen im 1. Tertial, 7,10 (±4,00) Wochen im 2. Tertial sowie 6,54 (±4,63) Wochen im 3. Tertial (*p* = 0,048). Eine Übersicht der Arbeitsunfähigkeitsdauer in den einzelnen Tertialen ist in Abb. 1 dargestellt.

### Einschätzungen der Pandemieauswirkungen durch die Patienten

**Figure 2:**
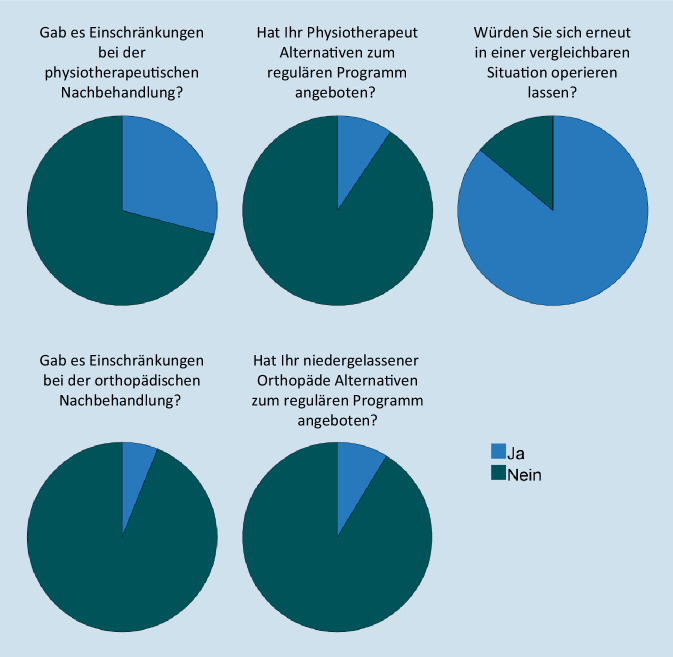

Von den 2020 operierten Patienten fühlten sich 39,8 % in der physiotherapeutischen und 8,5 % in der ärztlichen Nachbehandlung durch die COVID-19-Pandemie eingeschränkt (Abb. 2). Alternative Behandlungsoptionen wurden in der Physiotherapie 13,3 % der Patienten sowie in der Orthopädie 12,2 % der Patienten angeboten. 84 % der Patienten würden sich erneut während einer vergleichbaren Situation operieren lassen.

## Diskussion

Bei der vorliegenden Studie handelt es sich unseres Wissens um die erste Analyse der Auswirkungen der COVID-19-Pandemie auf die physiotherapeutische und ärztliche Nachsorge aus Patientensicht. Durchgeführt wurde die Befragung an Patienten nach einer Rekonstruktion des VKB. Diese Operation bietet in unseren Augen optimale Voraussetzungen für die Durchführung einer solchen Studie, da die Nachbehandlung stark standardisiert abläuft, was eine hohe Vergleichbarkeit sicherstellt. Zudem konnte für Rekonstruktionen des VKB bereits der hohe Stellenwert der physiotherapeutischen postoperativen Nachbehandlung für das langfristige Operationsergebnis belegt werden [7, 9–11]. Dennoch wollen wir an dieser Stelle betonen, dass sich ein großer Teil der Ergebnisse vermutlich auch auf andere Erkrankungen übertragen lässt, bei denen eine enge ärztliche und physiotherapeutische Nachbehandlung notwendig ist. Die Rekonstruktion des VKB wurde für die vorliegende Studie aus den o. g. Gründen stellvertretend als Beispiel gewählt. Insgesamt konnten wir zeigen, dass im Jahr 2020 im Zusammenhang mit der COVID-19-Pandemie die Wartezeiten auf Termine signifikant zunahmen, signifikant mehr Termine abgesagt wurden und es eine deutliche Tendenz zu einem verminderten Bewegungsumfang 3 Monate postoperativ gab. Gleichzeitig konnte dennoch durch eine Ausweitung der Möglichkeiten zur Arbeit im Homeoffice die Dauer der postoperativen Arbeitsunfähigkeit bei vielen Patienten reduziert werden.

Hinsichtlich der physiotherapeutischen Nachsorge berichteten die Patienten von einer signifikant längeren Wartedauer auf Termine sowie auch einer höheren Anzahl abgesagter Termine und vergeblicher Terminanfragen. Folgerichtig fand auch der erste postoperative Termin zur Physiotherapie signifikant später statt (1,15 Wochen 2019 vs. 1,88 Wochen 2020). Dies bestätigt den allgemeinen Eindruck, dass die COVID-19-Pandemie einen relevanten Einfluss auf die postoperative Nachsorge hatte, obwohl Physiotherapie auch während aller „Lockdown“-Phasen als Teil der Grundversorgung weiter in Anspruch genommen werden konnten. Eine mögliche Erklärung für diese Auswirkungen bietet eine Onlineumfrage mit 1370 Therapeuten in der ambulanten Heilmittelversorgung, in der 88 % der Teilnehmer eine höhere psychische Belastung aufgrund der COVID-19-Pandemie angaben, insbesondere aufgrund des zeitlichen Drucks durch die Hygienemaßnahmen, wirtschaftlichen Sorgen und Angst vor Infektion Angehöriger [12]. Interessanterweise steigerte sich die durchschnittliche Wartedauer auf Termine von 1,09 Wochen auf 2,57 Wochen um mehr als das doppelte, wohingegen es beim Zeitpunkt des ersten postoperativen Termins zu einer etwas geringeren Änderung von 1,15 auf 1,88 Wochen kam. Dies lässt vermuten, dass einige Patienten entsprechende Schwierigkeiten bereits vorausgesehen und sich daher früher als üblich um Termine gekümmert hatten.

Auch wenn bislang keine weitere Studie zu den Auswirkungen auf die physiotherapeutische Nachsorge nach VKB-Rekonstruktion in Deutschland vorliegt, bestätigen die international zu ähnlichen Fragestellungen veröffentlichten Studien diesen Eindruck, teils mit noch deutlicheren Unterschieden. So berichtet eine indische Studie, dass dortige Physiotherapeuten die Anzahl ihrer Patienten und die durchschnittliche Länge der Therapiesitzungen reduzieren mussten und sich durch das permanente Tragen der Schutzkleidung und die Infektionsangst physisch und psychisch beeinträchtigt fühlten [13]. In einer weiteren Studie aus Portugal bei 619 Physiotherapeuten gaben 73,2 % an, dass sie pandemiebedingt keinen direkten Patientenkontakt mehr hatten und sich stattdessen oft für eine Therapie aus der Distanz mit schriftlichen Behandlungsverordnungen und synchroner Behandlung per Videokonferenz entschieden [13]. Im Vergleich dazu scheinen die Auswirkungen in Deutschland eher gering geblieben zu sein, was sich auch in der trotz der Einschränkungen hohen Zufriedenheit (2020 im Durchschnitt 7,8/10) niederschlägt.

Insgesamt sehr ähnliche Ergebnisse zeigten sich in unserer Studie hinsichtlich der ärztlichen Nachbehandlung. Auch hier kam es zu einer signifikanten Zunahme der Wartedauer auf Termine sowie der Anzahl vergeblicher Terminanfragen und abgesagter Termine mit einer entsprechenden Verzögerung des ersten postoperativen Nachsorgetermins. Im Gegensatz zur Physiotherapie gab es hier nur eine Tendenz zu etwas geringerer Zufriedenheit ohne signifikanten Unterschied zwischen 2019 und 2020 (7,48/10 vs. 7,16/10, *p* = 0,4). Eine mögliche Erklärung hierfür ist der insgesamt deutlich intensivere Patientenkontakt während der physiotherapeutischen Behandlung, sodass Einschränkungen hier noch deutlicher wahrgenommen werden. Dazu passend gaben nur 8,5 % der Patienten an, sich bei der ärztlichen Nachbehandlung deutlich eingeschränkt gefühlt zu haben, wohingegen 39,8 % deutliche Einschränkungen in der physiotherapeutischen Nachsorge verspürten.

Neben den subjektiven Angaben der Patienten haben wir unseres Wissens als erste Studie auch die Auswirkungen auf das klinische Ergebnis in Form des postoperativen Bewegungsumfangs nach 3 Monaten untersucht. Hierbei zeigte sich bei den 2020 operierten Patienten eine deutliche Tendenz zu einem häufigeren Auftreten postoperativer Bewegungsdefizite gegenüber 2019, welche allerdings nicht signifikant war (Beugung: 10 % vs. 23,3 %, *p* = 0,197; Streckung: 4,3 % vs. 18,8 %, *p* = 0,097). Auch wenn (mutmaßlich aufgrund des zu kleinen Patientenkollektivs) keine signifikanten Unterschiede gezeigt werden konnten, bestätigt die deutliche Tendenz unserer Meinung nach dennoch den hohen Stellenwert der postoperativen Nachsorge sowie die relevanten Auswirkungen der COVID-19-Pandemie.

Trotz der häufig geschilderten Einschränkungen in der Nachsorge gaben 34,9 % der 2020 operierten Patienten an, dass sich durch die im Rahmen der Pandemiebekämpfung erfolgte Ausweitung der Möglichkeiten zur Arbeit im Homeoffice die Dauer der Arbeitsunfähigkeit verkürzte. Im Vergleich mit den 2019 operierten Patienten gab es 2020 dennoch über das gesamte Jahr betrachtet eine Tendenz zu einer längeren Arbeitsunfähigkeit (7,77 Wochen vs. 8,53 Wochen, *p* = 0,688). Eine mögliche Erklärung bietet die genauere Betrachtung der Arbeitsunfähigkeitsdauer in den einzelnen Tertialen. Während es im ersten Tertial 2020 zu einem deutlichen Anstieg von 7,77 auf 13,04 Wochen kam, fiel die durchschnittliche Dauer bereits ab dem 2. Tertial mit 7,1 Wochen wieder deutlich ab und hielt sich auch im 3. Tertial mit 6,54 Wochen auf einem ähnlichen Niveau. Auch die einfaktorielle Varianzanalyse bestätigte signifikante Unterschiede in der Dauer der Arbeitsunfähigkeit zwischen den Tertialen (*p* = 0,048). Der Grund für diesen zeitlichen Verlauf ist wahrscheinlich darin begründet, dass es zu Beginn der Pandemie recht schnell zu deutlichen Einschränkungen in der Nachbehandlung mit entsprechend verlängerter Krankheitsdauer kam, wohingegen Arbeitgeber erst mit einer gewissen Latenz auf Angebote wie Homeoffice umstellten. Der hohe Anteil an Patienten, die eine Verkürzung der Arbeitsunfähigkeit angaben, zeigt eindrucksvoll das Potenzial solcher nun erstmalig großflächig eingeführten Maßnahmen.

In der physiotherapeutischen und ärztlichen Nachbehandlung hingegen wurden den Patienten nur relativ selten (Physiotherapie: 13,3 %; Orthopädie: 12,2 %) aufgrund der Pandemie Alternativen zum regulären Programm (z. B. Onlinetermine, Telefontermine) angeboten. Hier bieten sich daher mögliche Ansatzpunkte, um die Auswirkungen im Falle erneuter vergleichbarer Situationen weiter zu reduzieren. Dass hier noch dringender Nachholbedarf besteht, zeigt sich auch darin, dass sich immerhin 8,4 % der 2020 operierten Patienten aufgrund der pandemiebedingten Einschränkungen nicht erneut in einer vergleichbaren Situation operieren lassen würden.

Trotz der eindeutigen Ergebnisse unserer Studie sollten bei der Interpretation die Limitationen aufgrund des Studiendesigns beachtet werden. So handelt es sich um eine retrospektive Studie mit den üblichen Einschränkungen, wobei die beiden verglichenen Studiengruppen recht unterschiedlich große Kohorten beinhalten. Eine Vergrößerung der 2019er-Kohorte wäre allerdings nur durch den zusätzlichen Einschluss noch früher operierter Patienten möglich gewesen, was durch Abfrage weiter zurückliegender Erinnerungen unvermeidlich mit einer reduzierten Verlässlichkeit der Angaben verbunden gewesen wäre. Zudem unterliegen die Einschätzungen der Patienten zu großen Teilen keinen objektiven Kriterien, sondern vorwiegend subjektiven Wahrnehmungen. Die unterschiedlichen Kohortengrößen führen in erster Linie zu einer reduzierten statistischen Power, sodass eventuell nicht alle tatsächlich bestehenden Unterschiede zwischen den Gruppen tatsächlich nachgewiesen werden konnten, was z. B. auf unsere Beobachtungen zum Bewegungsdefizit zutreffen könnte.

## Fazit für die Praxis

- In unserer Studie konnten wir zeigen, dass es durch die COVID-19(„coronavirus disease“ 2019)-Pandemie und die dadurch bedingten Maßnahmen zur Kontaktbeschränkung zu signifikanten Einschränkungen sowohl in der physiotherapeutischen als auch in der ärztlichen Nachsorge gekommen ist, obwohl diese als Teil der Grundversorgung nie von einem „Lockdown“ betroffen waren.
- Diese Einschränkungen schlugen sich auch in einer Tendenz zu häufigerem Auftreten von Beuge- und Streckdefiziten 3 Monate postoperativ nieder, auch wenn die Unterschiede nicht signifikant waren.
- Es besteht daher Bedarf an einem weiteren Ausbau alternativer Therapieoptionen, die bislang nur von ca. 13 % der physiotherapeutischen und ärztlichen Praxen angeboten werden.
- Das große Potenzial solcher Angebote zeigt der große Erfolg der bisherigen Maßnahmen wie Homeoffice, durch die über ein Drittel der 2020 operierten Patienten die Dauer ihrer Arbeitsunfähigkeit subjektiv verkürzen konnten.
- Die vorliegende Untersuchung anhand von Rekonstruktionen des vorderen Kreuzbands sollte beispielhaft für eine Vielzahl an Erkrankungen gesehen werden, für die ähnliche Auswirkungen zu vermuten sind.

## Abkürzungen

ANOVA: Varianzanalyse
COVID-19: „Coronavirus disease“ 2019
VKB: Vorderes Kreuzband
